# Role of CD1d- and MR1-Restricted T Cells in Asthma

**DOI:** 10.3389/fimmu.2018.01942

**Published:** 2018-08-28

**Authors:** Chiaki Iwamura, Toshinori Nakayama

**Affiliations:** ^1^Division of Immunology, Boston Children's Hospital, Boston, MA, United States; ^2^Department of Immunology, Graduate School of Medicine, Chiba University, Chiba, Japan

**Keywords:** CD1d, MR1, asthma, invariant NKT (iNKT) cells, mucosal-associated invariant T (MAIT) cells

## Abstract

Innate T lymphocytes are a group of relatively recently identified T cells that are not involved in either innate or adaptive immunity. Unlike conventional T cells, most innate T lymphocytes express invariant T cell receptor to recognize exogenous non-peptide antigens presented by a family of non-polymorphic MHC class I-related molecules, such as CD1d and MHC-related molecule-1 (MR1). Invariant natural killer T (iNKT) cells and mucosal-associated invariant T (MAIT) cells quickly respond to the antigens bound to CD1d and MR1 molecules, respectively, and immediately exert effector functions by secreting various cytokines and granules. This review describes the detrimental and beneficial roles of iNKT cells in animal models of asthma and in human asthmatic patients and also addresses the mechanisms through which iNKT cells are activated by environmental or extracellular factors. We also discuss the potential for therapeutic interventions of asthma by specific antibodies against NKT cells. Furthermore, we summarize the recent reports on the role of MAIT cells in allergic diseases.

## Introduction

Innate-like T cells (CD1-restricted T cells or MHC-related molecule-1 (MR1)-restricted T cells) are classified as innate lymphoid cells that have features similar to those of the cells involved in acquired immunity, such as T cell receptor (TCR) expression (1). However, their TCR repertoire is very limited, and they recognize self or exogenous non-peptide antigens presented by a family of non-polymorphic and MHC class I-related molecules (1).

NKT cells are characterized by the expression of TCRs with a limited repertoire, consisting of Vα14 and Jα18 (in mice) or Vα24 and Jα18 (in humans) (2). In addition, their sets of Vβs are also skewed toward mainly Vβ8.2 (in mice) and Vβ11 (in humans). Since NKT cells have limited TCRs, they are called invariant natural killer T (iNKT) cells. α-galactosylceramide (α-GalCer) presented by CD1d is the most potent and well-analyzed ligand that activates iNKT cells (2). Activated iNKT cells regulate various immune responses to protect us from tumors or infectious diseases (3, 4). However, these cells can also contribute to chronic inflammatory disease, such as allergic inflammation and autoimmune responses (5, 6).

Like iNKT cells, mucosal-associated invariant T (MAIT) cells express a semi-invariant TCR with a unique TCRα chain (Vα19-Jα33 in mice, Vα7.2-Jα33 in humans) and a restricted set of TCRβ chains (7). MAIT cells are activated by a bacterial riboflavin derivative presented by MR1 (8). Although MAIT cells have been suggested to play a role in antibacterial immunity through sensing MR-1-bound microbial products, it has been speculated that these cells may also be involved in regulating beneficial host commensal interactions in the intestine and potentially in the lung (9, 10). As well as participating in antimicrobial immunity, MAIT cells may be involved in the control of chronic inflammation (11).

Another interesting feature of these MAIT and iNKT cell populations is their memory-like phenotype (12). They are able to produce effector cytokines, cytolytic molecules, and growth factors at early time points of immune responses. Therefore, they are considered to play an essential role in the host defense immune responses. Both populations have distinct and characteristic tissue localization, as NKT cells reside in the thymus, spleen, lung and liver, while MAIT cells are preferentially found in the gut lamina propria, lung and liver (13, 14). NKT cells are relatively abundant in mice and show a lower frequency in humans, whereas the opposite situation is true for MAIT cells (15).

Upon activation, iNKT cells produce a large amount of both Th1 and Th2 cytokines in addition to inflammatory cytokines, such as interleukin (IL)-17 and tumor necrosis factor α (TNFα). iNKT cells show heterogeneity in their transcriptional factors. Three different subsets of iNKT cells have been shown to produce distinct cytokines, defined as NKT1, NKT2 and NKT17 (16, 17). iNKT cells are also distinguished by their surface molecules, such as CD4 and IL-17RB. CD4^+^IL-17RB^+^ iNKT cells in particular produce IL-13, IL-9, IL-10, IL-17A, and IL-22 (18). Since these cytokines exert different immunoregulatory functions, certain populations of iNKT cells might contribute to the development of chronic disorders, such as allergic diseases (18). MAIT cells produce IFN-γ, IL-4, IL-17, and TNFα (12, 19). Lepore et al. suggested that IL-5 and IL-13 can be produced by MAIT clones (20). It would be possible to identify distinct subsets of MAIT cells, that can produce specific cytokines such as is observed in the case of iNKT cells.

Asthma is a chronic inflammatory disease in the lung that causes recurring periods of wheezing, chest tightness, shortness of breath and coughing. It is well established that allergic asthma is induced by Th2 cell-mediated immune responses (21). Studies by our group and other authors have revealed that chronic airway inflammation in asthma patients is caused by pathogenic memory Th2 cells, which express high levels of IL-33 receptor ST2 and have a CD161^high^CRTH2^high^ phenotype in human (22–25).

Memory T cells are considered to play a beneficial role by responding immediately and strongly to the secondary invasion by the same antigen of a microorganism (26). However, memory T cells can induce adverse effects in cases of chronic inflammatory disease if they respond to allergens or self-antigens repeatedly for a long duration (27). Thus, allergen-specific memory Th2 cells, particularly the pathogenic subpopulation of ST2^+^ Th2 cells paly important roles in the pathogenesis of IL-5-induced eosinophilic inflammation and fibrotic responses (24, 28). However, it is also recognized that asthmatic patients show heterogeneous phenotypes, including so-called type-1 and type-2 mixed inflammation with neutrophilic infiltration. Thus, other cell types, such as NKT cells likely contribute to the development or exacerbation of asthma (29).

This review describes and discusses the immunoregulatory roles of innate-like T cells in asthma in animal models and human patients.

## Beneficial and detrimental effects of iNKT cells for allergic asthma

Many investigators have tried to determine the roles of iNKT cells in asthma over the past 20 years. To this end, Akbari et al. assessed OVA-induced airway hyperactivity (AHR) and allergic airway inflammation in iNKT cell-deficient Jα281 knockout (KO) and CD1d KO mice (30). They noted a significant defect in the development of AHR and inflammation in these NKT cell deficient mice. The defects were corrected by the adoptive transfer of iNKT cells in an IL-4- and IL-13-dependent manner. Therefore, iNKT cells were considered to contribute to the development of AHR and airway inflammation independent of Th2 cells. In addition, the same group showed that non-classical NKT cells, which are restricted to a β2-microgloblin-independent form of CD1d, also contribute to the development of AHR (31). Woo et al. further suggested that iNKT cells are also required for the generation of Th2 cells by recruiting CD103^+^ dendritic cells (DCs) to the lung via the XCL1-XCR1 axis (32). Furthermore, another group suggested that iNKT cells act as an adjuvant to enhance allergic asthma, as systemic iNKT cell activation by α-GalCer administration or adoptive transfer of iNKT cells before OVA challenge significantly augmented the Th2 inflammatory responses (33). These results indicate that iNKT cells have detrimental effects in allergic asthma.

Simultaneously, other groups reported experimental results indicating that iNKT cells are not involved in the development of allergic asthma. OVA-induced allergic inflammation was not reduced in CD1d-deficient mice or β2-microgloblin KO mice lacking iNKT cells (34, 35). Moreover, a protective role of iNKT cells in allergic asthma was suggested. Subsequent AHR in these models can be suppressed by the systemic activation of iNKT cells by α-GalCer treatment or the transfer of α-GalCer-loaded bone marrow-derived DCs before OVA challenge in an IFN-γ-dependent manner (36, 37). In addition, Grela et al. reported that IFN-γ-producing iNKT cells stimulated with toll like receptor (TLR) 7 agonist (R848) attenuated allergic asthma, which is consistent with the finding that TLR7 stimulation not only enhances viral responses but also alleviates experimental asthma (38).

Thus, iNKT cells display either beneficial or detrimental effects in allergic asthma. These conflicting effects may be due to the various cytokine production patterns of iNKT cells under different conditions. IL-4 or IL-13 production from iNKT cells is required for the development of allergic asthma in mouse models, while iNKT cells can produce IFN-γ, which can suppress the Th2 response and thereby prevent allergic asthma. However, even when employing similar protocols, different institutes obtained completely different findings (33, 36, 37). Since iNKT cells can detect bacterial components through their invariant TCRs or Toll-like receptors, the difference in the lung microbiota may affect the function of distinct iNKT cell subsets, such as NKT1 and NKT2.

Although inducing Th1 bias by iNKT cell activation may result in the inhibition of AHR and eosinophilic infiltration, our recent study shed light on how NKT cell activation can suppress Th2 type inflammation. While immunological memory plays a central role in providing protection against infection or cancer, antigen-specific memory CD4 T cells contribute to the pathogenesis of allergic and autoimmune disorders by recognizing allergens or self-antigens (24, 39). Our data showed that the activation of iNKT cells with α-GalCer during the memory phase resulted in the downregulation of IL-4, IL-5, and IL-13 and up-regulation of IFN-γ in memory Th2 cells (40). These functionally altered memory Th2 cells display a decreased capability to induce Th2 cytokines and eosinophilic airway inflammation. We therefore concluded that activated iNKT cells directly regulate memory Th2 cell function *in vivo*. Chang et al. showed another inhibitory mechanism for allergic disorder by iNKT cells. They found that influenza infection in neonates helped prevent allergic asthma by inducing CD4^neg^CD8^neg^ iNKT cell activation, which is associated with the expansion of regulatory T cells (41). The inhibitory effect required T-bet and TLR7 expression in iNKT cells. Furthermore, the administration of α-GalCer or glycolipid derived from *Helicobacter pylori* to neonates recapitulated the result (41), suggesting that infection with certain microorganisms can prevent the subsequent development of allergic asthma by expanding a specific subset of iNKT cells. Therefore, the authors proposed that treatment of children or allergic patients with compounds such as α-GalCer or other glycolipids derived from microorganisms might be effective in preventing or improving the development or symptoms of allergic asthma.

## Lung iNKT cell-dependent allergic or non-allergic asthma

**Figure 1:**
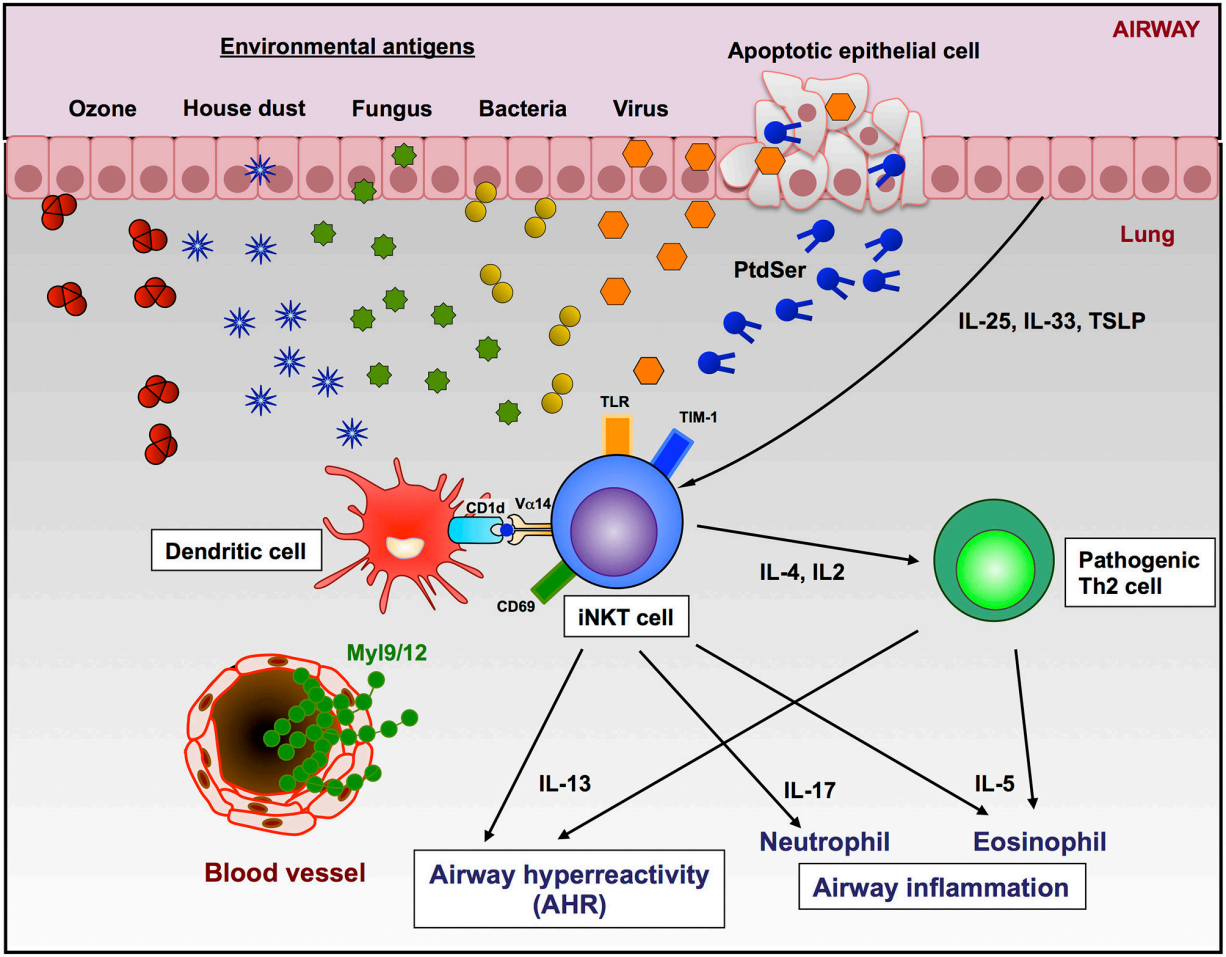
Roles of iNKT cells and Th2 cells in the development of AHR and airway inflammation. Lung iNKT cells can be activated by environmental substances in a TCR-CD1d-dependent manner or extracellular factors (cytokines, TLR ligands, or apoptotic cells by virus infection). The CD69-Myl9 system may regulate the infiltration of iNKT cells into inflamed tissues through blood vessels. The activation of lung iNKT cells resulted in AHR and infiltration of either neutrophils, eosinophils, or both in the airway by producing cytokines.

Lung iNKT cells are relatively abundant compared to iNKT cells in the peripheral blood (14). The activation of pulmonary iNKT cells by the intranasal α-GalCer administration rapidly induced AHR and eosinophilic inflammation in naïve mice, and this effect was independent of conventional CD4 T cells (42). Michel et al. showed that NK1.1^neg^ iNKT cells produced high levels of IL-17 and induced neutrophilic infiltration following the intranasal administration of α-GalCer in a murine model (43). In addition, the development of AHR was observed in non-human primates by the direct activation of pulmonary iNKT cells with α-GalCer, indicating that pulmonary iNKT cells are critical effector cells in these animal models (44). Our previous study showed that α-GalCer induced AHR and neutrophilic infiltration, and the neutrophilic infiltration was significantly attenuated in CD69-deficient mice, indicating that activated iNKT cells-mediated asthmatic responses were dependent on CD69 expression (5). We recently identified myosin light chain (Myl) 9 and Myl12 as functional ligands for CD69 (45). We also showed that the interaction between CD69 on Th2 cells and Myl9 expressed on the luminal side of endothelial cells in the blood vessels recruits activated Th2 cells to the inflammatory site, resulting in airway inflammation (45, 46). CD69 on iNKT cells might therefore induce the migration of iNKT cells to the lung by binding to Myl9 or Myl12 and also play a critical role in the development of AHR and airway inflammation (Figure 1).

Even if iNKT cell activation in the lung does contribute to asthma, we are unlikely to be exposed to α-GalCer, a component of marine sponge, in our daily lives. Several studies have indicated that substances naturally existing in our environment, such as allergens, pathogens and air pollution, might activate iNKT cells and cause or exacerbate airway inflammation. Glycolipids from bacteria, such as *Sphingomonas, Borrelia*, and *Leishmania* species, are recognized by invariant TCR of iNKT cells (47). In particular, glycolipids purified from *Sphigomona* cell walls were shown to induce rapid AHR after respiratory administration in wild-type mice but not iNKT-deficient mice (42). Although a glycolipid that can induce iNKT cell activation has not been identified in viruses, Kim et al. suggested that viruses may facilitate CD1d antigen presentation and induce iNKT cell activation in an indirect manner (48). The authors also showed that IL-13 production from macrophages stimulated by iNKT cells during respiratory virus infection induces the development of AHR and mucus production independent of the adaptive immune response. *Aspergillus fumigatus* is a saprophytic fungus that is ubiquitous in the environment and is commonly associated with allergic asthma (49). Albacker et al. reported that the *Aspergillus funmigatus*-derived glycosphingolipid asperamide B directly activates iNKT cells in a CD1d-restricted, Myd88-independent, and dectin-1-independent manner (50). The intranasal administration of asperamide B rapidly induced AHR and neutrophil infiltration into the lung, suggesting that fungi can contribute to the induction of asthmatic symptoms by iNKT cells. Therefore, iNKT cells activated by glycolipids from microorganisms may contribute to the development and exacerbation of asthma symptoms in humans.

It was recently revealed that non-glycolipid stimulation could also activate iNKT cells, resulting in the induction of AHR. House dust extract (HDE) contains antigens and is capable of inducing airway inflammation by activating mouse Vα14 or human Vα24 NKT cells (51). The stimulation of mouse Vα14 iNKT cells was shown to be CD1d-dependent and not dependent on TLR agonist present in HDE. Although the antigen in HDE remains incompletely characterized, the authors suggested that the immunostimulatory material in HDE was of neither bacteria nor glycolipid origin (51). Ozone is an air pollutant that has also been reported to be associated with asthma (52, 53). The development of AHR was found to be inducible even in healthy individuals following exposure to ozone, which causes airway epithelial damage, and increased numbers of neutrophils (54, 55). Furthermore, asthmatic patients are more susceptible to the detrimental effects of this pollutant. A murine model of ozone induced-asthma revealed the indispensable role of IL-17-producing iNKT cells for the induction of AHR (56). Although how ozone activates iNKT cells is unclear at present, NKT cells activated by ozone can induce a form of asthma that is characterized by cellular infiltration and AHR.

In addition to the naturally existing molecules in the environment, extracellular factors are also known to activate iNKT cells. T cell immunoglobulin and mucin domain-1 (TIM-1) is an important asthma susceptibility gene and also a receptor for phosphatidylserine (PtdSer) (57), an important marker of cells undergoing programed cell death or apoptosis (58). NKT cells can activate, proliferate, and produce cytokines through recognition of PtdSer by TIM-1 (59). Furthermore, the apoptosis of airway epithelial cells activates pulmonary NKT cells, resulting in AHR and suggesting that TIM-1 serves as a pattern recognition receptor on NKT cells that senses PtdSer on apoptotic cells as a damage-associated molecular pattern (60). Previous studies have shown that apoptosis induced by virus infection or ozone exposure can trigger NKT activation (48, 56, 60), as infection with some viruses triggers apoptosis and externalization of PtdSer. In addition, it has been reported that TLR signaling enhances the activation of iNKT cells. Vultaggio et al. showed that systemic dsRNA (poly (I:C)) selectively upregulates the IL-17 production from iNKT cells activated by α-GalCer. The authors therefore expected that the exacerbation of airway inflammation might be induced by certain virus infections (61). Furthermore, several cytokines involved in the initiation and amplification of Th2 responses have been reported (62). IL-25 is capable of enhancing AHR and is produced by activated Th2 cells, epithelial cells, basophils, and mast cells (63). The administration of recombinant IL-25 induced Th2-type responses, including increased serum IgE levels, eosinophilia, pathological changes in the lung, and AHR. These symptoms induced by IL-25 were not observed in iNKT cell-deficient mice (64, 65). Moreover, iNKT cells expressing IL-17 receptor B were shown to be essential for IL-25-induced AHR using an adoptive transfer model (65). Thymic stromal lymphoprotein (TSLP) is also considered to play an important role in the iNKT cell-dependent asthma model (66). While the targets of TSLP are T cells, mast cells, basophils, and DCs, Nagata et al. demonstrated that TSLP also acts on iNKT cells to enhance AHR by up-regulating their production of IL-13 (67). IL-33 enhanced the production of Th1 and Th2 cytokines in activated NKT cells (68, 69). These results indicate that natural ligands in the environments act as antigens for iNKT cells to induce allergic asthma, and TCR-independent stimuli to iNKT cells may exacerbate the asthmatic symptoms such as AHR (Figure 1).

Although it is obvious that the direct activation of lung iNKT cells causes lung inflammation, which types of inflammation are induced is still controversial. Two groups claimed that the intranasal administration of α-GalCer induced allergic airway inflammation because eosinophil infiltration into the lung, a feature of type 2-mediated responses, was observed in IL-4- and IL-13-dependent manners (42, 70, 71). However, neutrophil infiltration, which represents non-allergic airway inflammation, is frequently observed in severe or Th17-mediated asthma (72, 73). The activation of iNKT cells by the intranasal administration of α-GalCer, asperamide B or PtdSer induces pulmonary neutrophil infiltration, suggesting that iNKT cell may contribute to non-allergic airway inflammation (5, 43, 50, 59). In contrast, equivalent numbers of eosinophils and neutrophils have been noted with ozone or poly (I:C) stimulation (56, 61). This discrepancy in outcomes may be due to the activation of distinct subsets of iNKT cells: one produces IL-13 and IL-5, which activate and recruit eosinophils; the other produces IL-17, thereby inducing the recruitment of neutrophils. Additional flow cytometry single cell analyses addressing the precise production profiles of cytokines in iNKT cells are needed in order to discriminate the infiltrated subsets.

With many clinical and experimental examinations, it has been revealed that asthma is more heterogeneous and complex than previously thought. While allergic asthma is induced by allergens and mediated by Th2 cells, a non-allergic form of asthma is caused independent of Th2 responses (29). Non-allergic asthma is induced by multiple environmental factors, such as air pollution (smoke, ozone, and diesel particles) and virus infection. Although the immunological pathways of non-allergy asthma are still unclear, the activation of iNKT cells with their specific ligands or cytokines may contribute to the development of non-allergy asthma.

Taken together, these findings suggest that different types of iNKT cell ligands may activate distinct subsets of iNKT cells, thereby resulting in distinct patterns of airway inflammation. Therefore, lung iNKT cell activation may contribute to the development of various types of asthmatic inflammation (Figure 1).

## Therapeutic intervention for iNKT cell-dependent allergic asthma

As we pointed out above, iNKT cells may play have a critical role in the development or exacerbation of asthma. Although further investigations are needed, Dimaprit (H2 histamine receptor agonist) or intravenous immunoglobulin treatment does appear to suppress iNKT cell-dependent allergic asthma (74, 75). The administration of anti-mouse CD1d monoclonal antibodies (20H2) or CD1d-dependent antagonist has also been shown to suppress OVA-induced AHR and inflammation in murine models (76, 77). Indeed, McKnight et al. reported that anti-mouse CD1d monoclonal antibody (20H2) treatment before the intranasal administration of α-GalCer impaired iNKT cell-induced AHR in an experimental mouse model of asthma, while this antibody did not suppressed OVA-induced allergic asthma. These results suggest that this antibody may attenuate non-allergic asthma (35). Anti-human CD1d antibody (NIB.2) possesses a high affinity for human and cynomolgus macaque CD1d and inhibits NKT cell activation by inhibiting the interactions of the TCRβ chain of iNKT cells with CD1d (78). NIB.2 treatment significantly reduced the cytokine levels and numbers of lymphocytes and macrophages in the bronchoalveolar lavage fluid (BALF) in a primate model of asthma (78). However, this antibody may affect other CD1d-restricted T cells that are not involved in airway inflammation (79). Therefore, the development of a more specific method will pave the way for therapeutic interventions to alleviate symptoms.

Mouse invariant monoclonal antibody, NKT14 was found to specifically bind to invariant TCR of mouse iNKT cells and deplete iNKT cells in mice via antibody-dependent cellular cytotoxicity and complement-dependent cytotoxicity for 3 weeks (80). The elimination of iNKT cells was sufficient to prevent murine AHR and pulmonary eosinophilic inflammation elicited by the oropharyngeal inhalation with α-GalCer (71). In addition, NKT14 administration prior to sensitization abrogated either antigen-mediated AHR alone or both AHR and pulmonary inflammation (71, 80).

## Role of iNKT cells in asthma patients

In order to determine the role of iNKT cells in human asthma, many investigators have examined iNKT cells in asthma patients with regard to their numbers and the production of cytokines (Table 1). An initial report published in 2006 by Akbari et al. stated that more than 60% of CD4 T cells in the BALF from severe asthmatic patients were iNKT cells, while the infiltration of iNKT cells was not observed in patients with other pulmonary diseases, such as sarcoidosis, or in healthy controls (81). Three other supportive reports showed that asthmatic patients display higher frequency of iNKT cells in BALF as compared to healthy control donors do (82, 83, 91). However, the very high numbers of iNKT cells (~60% among CD4 T cells) reported by Akbari et al. have not been replicated by other investigators.

**Table 1:** iNKT cells in patients with asthma.

	**Frequency of iNKT cells are**									
**Year**	**Increased compared to HCs**					**Decreased compared to HCs or comparable to HCs**				
	**Ref**	**Staining method**	**Sample**	**Type of patients**	**% of NKT**	**References**	**Staining method**	**Sample**	**Type of patients**	**% of NKT**
2006	Akbari et al. (81)	CD1d-restricted NKT cells	BALF	Healthy controls	0.9% of CD3^+^ cells (mean)					
				Patients with asthma treated with corticosteroids	73% of CD3^+^ cells (mean)					
				Patients with asthma not treated with corticosteroids	72% of CD3^+^ cells (mean)					
	Hamzaoui et al. (82)	CD56^+^ Vα24^+^ NKT cells	Sputum	Healthy controls	0.09 ± 0.02% in sputum					
				Severe asthma	0.82 ± 0.07% in sputum					
				Mild asthma	0.07 ± 0.05% in sputum					
	Pham-Thi et al. (83)	CD1d-restricted NKT cells	BALF	Non-atopic controls	0.116 ± 0.03% in T cells					
				Asthma	0.435 ± 0.09% in T cells					
2007						Vijayanand et al. (84)	Vα24^+^ Vβ11^+^ NKT cells	Sputum	Control subjects	0–0.6% of CD3^+^ cells
									Subject with moderately severe asthma treated with corticosteroids	0–1.3% of CD3^+^ cells
									Subject with mild asthma not treated with corticosteroids	0–0.6% of CD3^+^ cells
							Vα24^+^ Vβ12^+^ NKT cells	BALF	Control subjects	Data not shown
									Subject with moderately severe asthma treated with corticosteroids	0–0.2% of CD3^+^ cells
									Subject with mild asthma not treated with corticosteroids	0% of CD3^+^ cells
							CD1d-restricted NKT cells	Sputum	Control subjects	0–0.1% of CD3^+^ cells
									Subject with moderately severe asthma treated with corticosteroids	0% of CD3^+^ cells
									Subject with mild asthma not treated with corticosteroids	0 of CD3^+^ cells
							CD1d-restricted NKT cells	BALF	Control subjects	Data not shown
									Subject with moderately severe asthma treated with corticosteroids	0–0.8% of CD3^+^ cells
									Subject with mild asthma not treated with corticosteroids	0 or 0.3% of CD3^+^ cells
							6B11^+^ NKT cells	Sputum	Control subjects	0–0.6% of CD3^+^ cells
									Subject with moderately severe asthma treated with corticosteroids	0 or 0.5% of CD3^+^ cells
									Subject with mild asthma not treated with corticosteroids	0% of CD3^+^ cells
							6B11^+^ NKT cells	BALF	Control subjects	Data not shown
									Subject with moderately severe asthma treated with corticosteroids	0–0.6% of CD3^+^ cells
									Subject with mild asthma not treated with corticosteroids	0% or 2.7% of CD3^+^ cells
						Mutalithas et al. (85)	6B11^+^ NKT cells	BALF	Controls	0.12 ± 0.02% of CD3^+^ cells
									Asthma	0.37 ± 0.1% of CD3^+^ cells
2009	Matangkas-ombut et al. (86)	CD1d-restricted NKT cells	BALF	No asthma	0.07–0.68% of CD3^+^ cells					
				Well controlled asthma	0.05–9.57% of CD3^+^ cells					
				Severe asthma	2.43–7.18% of CD3^+^ cells One patient shows 64.5% of iNKT cells					
2010						Brooks et al. (87)	6B11^+^ NKT cells	Sputum	Controls	Median of 0.06% (IQR, 0-0.2%) in all T cells
									Asthma	Median of 0.07% (IQR, 0-0.17%) in all T cells
	Koh et al. (88)	CD56^+^ NKT cells	Sputum	Controls	0.005 ± 0.005% in CD3^+^ cells	Koh et al. (88)	CD56^+^ NKT cells	PBMCs	Controls	1.81 ± 0.33% in CD3^+^ cells
				Asthma	0.27 ± 0.08% in CD3^+^ cells				Asthma	1.98 ± 0.37% in CD3^+^ cells
		6B11^+^ NKT cells	Sputum	Controls	0.32 ± 0.15% in CD3^+^ cells		6B11^+^ NKT cells	PBMCs	Controls	0.22 ± 0.05% in CD3^+^ cells
				Asthma	0.76 ± 0.3% in CD3^+^ cells				Asthma	0.24 ± 0.04% in CD3^+^ cells
		Vα24^+^ NKT cells	Sputum	Controls	0.03 ± 0.02% in CD3^+^ cells		Vα24^+^ NKT cells	PBMCs	Controls	0.42 ± 0.05% in CD3^+^ cells
				Asthma	0.14 ± 0.03% in CD3^+^ cells				Asthma	0.48 ± 0.04% in CD3^+^ cells
2012						Yan-Ming et al. (89)	Vα24^+^ Vβ11^+^ NKT cells	PBMCs	Controls	0.135 ± 0.061% in blood
									Asthma	0.051 ± 0.041% in blood
2014						Shim et al. (90)	Vα24^+^ Vβ11^+^ NKT cells	PBMCs	Controls	0.2 ± 0.1% in blood
									Asthma	0.2 ± 0.2% in blood
							6B11^+^ Vβ12^+^ NKT cells	PBMCs	Controls	0.07 ± 0.01% in blood
									Asthma	0.2 ± 0.1% in blood

*BALF, bronchoalveolar lavage fluid; PBMCs, peripheral blood mononuclear cells; HCs, healthy controls*.

In contrast, a similar study by other group found that the number of iNKT cells was not increased in patients with asthma (91). Another group reported that iNKT cells were found in low numbers in the sputum or BALF of patients with asthma, chronic obstructive pulmonary disease and healthy controls, with no significant differences among the three groups (84). Mutalithas et al. also reported similar results in the BALF (85). Furthermore, the influx of iNKT cells in the airways was not observed after segmental allergen challenge (92, 93). To explain this discrepancy, Thomas et al. (93) and Vijayanand (84) pointed out that 6B11 antibody was able to stain alveolar macrophages nonspecifically. They suggested that the higher frequency of iNKT cells was due to the non-specific binding to the cells, and that the lymphocyte population should be gated for the analysis of iNKT cells (83, 91). However, Akbari et al. argued that they had already gated the lymphocyte population and used a CD1d-tetramer instead of 6B11 antibody to stain iNKT cells. In addition, those authors readdressed the issue regarding the number of iNKT cells in BALF from patients with severe asthma the next year (86). They confirmed that patients with severe asthma had a significantly increased number of iNKT cells compared to healthy controls. In this report, however, CD1d-restricted iNKT cells accounted for 2–7% of total CD3^+^ cells in the BALF of asthmatic patients, and only 1 patient with severe asthma had an iNKT cell proportion of 64.5% (93). The findings of Reynolds et al. supported the increase in the number of iNKT cells in the lung using biopsies with allergen challenge (94). Nevertheless Brooks et al. subsequently suggested that the high frequency of iNKT cells detected in BALF was due to the non-specific staining of dead cells (87). In addition, they also indicated that there was no marked difference in the frequency of 6B11^+^ iNKT cells in sputum even when including dead cells in the samples.

After 2010, it was suggested that a reduced iNKT cell frequency in the PBMCs of asthmatic patients did not imply that iNKT cells were irrelevant to the development of asthma. Koh et al. showed that the numbers of NKT cells in peripheral blood did not differ markedly between patients and control groups (88). However, in sputum, the numbers of iNKT cells were significantly increased in patients with asthma. Their subsequent study demonstrated the negative correlation between blood iNKT cell number and eosinophils, cytokines, or chemokines in sputum (95). These results suggested that iNKT cell might be mobilized to the lung during the exacerbation. Two other groups also demonstrated the profound reduction or no increase in iNKT cells in the blood of asthma patients compared to the normal control group (89, 90). However, they also showed an increased IL-4 production in iNKT cells of asthma patients compared to controls. Pedroza showed that pediatric asthmatic patients undergoing exacerbations of asthma displayed increased numbers of iNKT cells in the blood that also produced less IFN-γ and more IL-4 than children with stable asthma or in healthy control children (96). These results suggest that Th2-like iNKT cells might be involved in the development of asthmatic exacerbations.

At present, studies on iNKT cells in asthma patients have provided conflicting results. The frequency of iNKT cells in the lungs is particularly hotly debated. As such, we conclude that the frequency of iNKT cell does not always reflect the severity of the diseases. Although there are some recent reports that suggest no correlation between the blood iNKT cell number and clinical asthma severity (97), it is becoming more widely recognized that iNKT cells likely play a role in the development and possibly exacerbation of allergic asthma. In addition, the studies of iNKT cells in other asthma etiologies, such as chronic, occupational, steroid-resistant, exercise-induced, and aspirin-induced asthma, where Th2 cells may not paly a major role, may provide new insights into these type of diseases. We therefore suggest a few experimental design approaches to adopt when studying the role of iNKT cells in particular diseases. First, in the flow cytometry analysis of iNKT cells in patients, lymphocytes, particularly live cells, should be gated for the analysis, and control staining, including with isotype controls, should be performed, with the results compared. This will prevent the contamination of cells with non-specific staining patterns. Second, more than two staining protocol should be employed. At least three different approaches have been established for identifying iNKT cells, such as CD1d-tetramer, anti-Vα24 antibody and 6B11 antibody recognizing the CDR3 region of Vα24-JαQ TCR. Although these approaches should theoretically provide similar results, using multiple staining protocols may help clear up any confusion if controversial results are obtained. Third, in addition to assessing the frequency of iNKT cells, their cytokine production (IL-2, IL-4, IFN-γ, or IL-17) should also be examined by flow cytometry. As we discussed above, it would be difficult to demonstrate the relevance of iNKT cells to diseases by analyzing only the frequency of such a small population. Examining changes in their function may therefore be useful for elucidating their contribution to the pathology of diseases.

## MR1-restricted cells

MAIT cells are a subset of innate-like T lymphocytes first described in 1999 (98). These MR-1-restricted cells are abundant in humans and can rapidly express a variety of pro-inflammatory cytokines (12). While iNKT cells are suggested to play critical roles in murine models of allergic airway diseases, they are rare in human airways. MAIT cells, by contrast, are 5- to 10-fold more abundant in humans than in mice (15). Since MAIT cells exist in the lung and may be able to produce Th2 cytokines (19, 20), these cells may contribute to the development of asthma. However, several reports have indicated a different role for these cells. Hinks et al. observed a striking deficiency of Vα7.2^+^ CD161^+^ T cells in blood, sputum, and bronchial biopsy samples, suggesting that the deficiency correlated with the severity of asthma (11, 99). A similar deficiency in humans was observed in autoimmune diseases (systemic lupus erythematosus, rheumatoid arthritis, Crohn's disease, ulcerative colitis, or chronic inflammatory disease, such as type 2 diabetes) (100–103). In addition, it was reported that an increased MAIT cell frequency at 1 year of age was associated with a decreased risk of asthma by 7 years of age (104). These results suggest that MAIT cells may play a protective role against chronic inflammation.

Given that MAIT cells respond to bacterial metabolites, it is possible that MAIT cell activation by gut or lung microbiota is required to prevent asthma. If MAIT cells can exert a suppressive function against chronic inflammation, this hypothesis would be inconsistent with their ability to produce various inflammatory cytokines. In addition, it was also reported that the numbers of MAIT cells producing IL-17 are increased in asthmatic patients (105). Since MAIT-deficient mice have been generated (106), investigations into the function of MAIT cells infiltrating the inflammatory site in mouse models may help provide answers.

## Conclusion

Studies investigating the roles of iNKT cells in allergic responses have helped to explain the Th2-dependent mechanisms underlying the development of allergic asthma. However, iNKT cells also have been suggested to be associated with the development of non-allergic airway inflammation that is induced and/or exacerbated by non-Th2 factors, such as viruses, air pollution and inflammatory cytokines (IL-17 or TNFα). Furthermore, recent studies have suggested that NKT cells or MAIT cells may play a critical role in the inhibition of asthmatic symptoms. Although a clear conclusion has not been reached due to inconsistent results, innate-like T cells apparently have critical and varied roles in regulating immune responses. As such, more intensive studies will be required in order to elucidate the mechanisms underlying the induction of various types of asthma by innate-like T cells and establish innovative therapeutic strategies.

## Author contributions

All authors listed have made a substantial, direct and intellectual contribution to the work, and approved it for publication.

### Conflict of interest statement

The authors declare that the research was conducted in the absence of any commercial or financial relationships that could be construed as a potential conflict of interest.

## Abbreviations

MR1: MHC-related molecule-1
TCR: T cell receptor
iNKT: invariant natural killer T
α-GalCer: α-galactosylceramide
MAIT: mucosal-associated invariant T
IL: interleukin
TNFα: tumor necrosis factor α
AHR: airway hyperactivity
KO: knockout
TLR: toll like receptor
Myl: myosin light chain
HDE: house dust extract
TIM-1: T cell immunogloblin and mucin domain-1
PtdSer: phosphatidylserine
TSLP: thymic stromal lymphoprotein
DCs: dendritic cells
BALF: bronchoalveolar lavage fluid.
